# Probing pattern and dynamics of disulfide bridges using synthesis and NMR of an ion channel blocker peptide toxin with multiple diselenide bonds†
†Electronic supplementary information (ESI) available: Details of synthesis and analytical characterization of the Sec-analog of [N17A/F32T]-AnTx mutant including quadrupole ESI and ESI-TOF MS spectra, overlay of ^1^H-^13^C HSQC spectra of the double mutant [N17A/F32T]-AnTx and its Sec-analog, scheme and Bruker code of the ^77^Se-decoupled ^1^H-^77^Se CPMG-HSQMBC pulse sequence and comparison of its performance to the classical HMQC method, ^1^H, ^13^C, ^15^N and ^77^Se resonance assignment of Sec-[N17A/F32T]-AnTx peptide. See DOI: 10.1039/c5sc03995a


**DOI:** 10.1039/c5sc03995a

**Published:** 2015-12-21

**Authors:** Krisztina Fehér, István Timári, Kinga Rákosi, János Szolomájer, Tünde Z. Illyés, Adam Bartok, Zoltan Varga, Gyorgy Panyi, Gábor K. Tóth, Katalin E. Kövér

**Affiliations:** a Department of Inorganic and Analytical Chemistry , University of Debrecen , Egyetem tér 1 , H-4032 , Debrecen , Hungary . Email: kover@science.unideb.hu; b Department of Organic and Macromolecular Chemistry , Ghent University , Kringslaan 281 S4 , 9000 , Ghent , Belgium; c Department of Medical Chemistry , University of Szeged , Dóm tér 8 , H-6720 , Szeged , Hungary . Email: toth.gabor@med.u-szeged.hu; d Department of Organic Chemistry , University of Debrecen , Egyetem tér 1 , H-4032 , Debrecen , Hungary; e Department of Biophysics and Cell Biology , University of Debrecen , Egyetem tér 1 , H-4012 , Debrecen , Hungary; f MTA-DE-NAP B Ion Channel Structure-Function Research Group , Egyetem tér 1 , H-4032 , Debrecen , Hungary; g MTA-DE Cell Biology and Signaling Research Group , University of Debrecen , Egyetem tér 1 , H-4032 , Debrecen , Hungary

## Abstract

Anuroctoxin (AnTx), a 35-amino-acid scorpion toxin containing four disulfide bridges, is a high affinity blocker of the voltage-gated potassium channel Kv1.3, but also blocks Kv1.2. To improve potential therapeutic use of the toxin, we have designed a double substituted analog, [N17A/F32T]-AnTx, which showed comparable Kv1.3 affinity to the wild-type peptide, but also a 2500-fold increase in the selectivity for Kv1.3 over Kv1.2. In the present study we have achieved the chemical synthesis of a Sec-analog in which all cysteine (Cys) residues have been replaced by selenocysteine (Sec) forming four diselenide bonds. To the best of our knowledge this is the first time to replace, by chemical synthesis, all disulfide bonds with isosteric diselenides in a peptide/protein. Gratifyingly, the key pharmacological properties of the Sec-[N17A/F32T]-AnTx are retained since the peptide is functionally active. We also propose here a combined experimental and theoretical approach including NOE- and ^77^Se-based NMR supplemented by MD simulations for conformational and dynamic characterization of the Sec-[N17A/F32T]-AnTx. Using this combined approach allowed us to attain unequivocal assignment of all four diselenide bonds and supplemental MD simulations allowed characterization of the conformational dynamics around each disulfide/diselenide bridge.

## Abstract 2

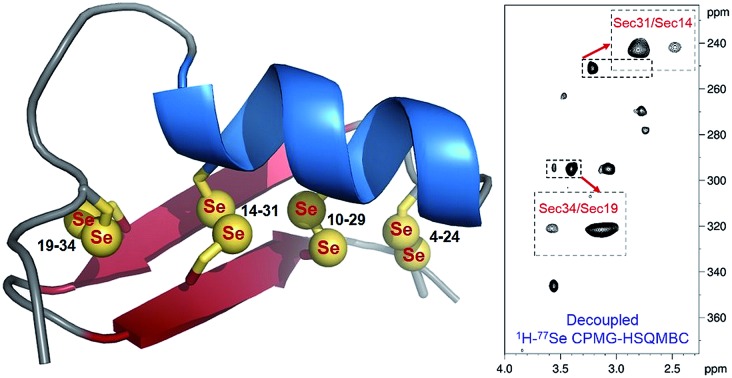
A biologically active peptide toxin containing four diselenide bonds was synthesized. The diselenide network and its dynamics were disclosed using a combined NMR and MD approach.

## Introduction

The disulfide pattern of polypeptides containing multiple disulfide bridges is of considerable interest because the presence of disulfide bonds is essential for maintaining the tertiary structure responsible for the observed biological activity. Therefore, a key issue in the structural characterization of these peptides is an unambiguous determination of the pattern of disulfide pairing. Since small, flexible disulfide-rich peptides are typically difficult to crystallize, therefore nuclear magnetic resonance (NMR) spectroscopy and mass spectrometry (MS) are the most commonly used techniques for elucidating their structures.^1^

MS determination of a disulfide bonding pattern is a challenging task primarily relying on manual interpretation.^2^,^3^ It requires either enzymatic digestion of the protein where disulfide scrambling may take place, or chemical modification if the target protein is cysteine rich, or else contains an unexpected folding pattern with unknown disulfide bonds.

NMR provides powerful tools for determining the three-dimensional structures of these small proteins and peptides, but, due to the unfavorable NMR properties of sulfur isotopes, disulfide bonds often present a ‘blind spot’ in NMR structural investigations.^1^ Experimental observations supported by DFT quantum-chemical calculations proved that the redox state of cysteine residues can be safely disclosed on the basis of Cys ^13^C_β_ chemical shifts.^4^ These data, however, do not provide information about the disulfide bridge connectivity. Heretofore the most commonly used approach for determining S–S connectivity relied on the detection of interresidue dipolar (NOE) interactions between the β-methylene protons of the covalently linked cysteine residues.^5^ Detection of these weak inter-disulfide NOE contacts is often hampered by spin-diffusion effects due to the strong intraresidue cross-relaxation between the geminal beta protons of Cys and/or in some cases by an enhanced conformational flexibility around the disulfide bonds, leading to an ambiguous assignment of the disulfide network. However, such uncertainties could be resolved by stereospecific deuterium labeling of the cysteine beta protons at the cost of laborious isotope labeling. Besides, in the case of closely packed disulfide bonds, the NOE contacts can be also ambiguous and lead to incorrect pairing of Cys residues. Alternatively, as has been recently suggested, replacement of the NMR-inactive ^32^S nucleus by ^77^Se possessing more favorable magnetic properties^6^,^7^ – by mutation of cysteine into selenocysteine (Sec) residues^8^–^13^ – may allow the detection of diselenide pairing directly through the three-bond ^3^*J*(^1^H, ^77^Se) couplings across the diselenide bond.^5^ Importantly, it was also confirmed that the isosteric replacement of the disulfide bond with diselenide has no significant effect, in general, on the three-dimensional structure and the associated biological activity of peptides.^14^ In addition, using ^77^Se NMR both the conformational and dynamic properties of diselenide (disulfide) bonds can be explored.

The Sec residues can be introduced into peptides or proteins either by chemical synthesis^11^,^12^,^14^–^16^ or by recombinant expression.^7^,^9^,^10^,^17^ Using chemical synthesis, substitution of only one or two disulfide bridges has been reported in most cases whereas multiple disulfide bonds can be replaced with diselenides by applying recombinant expression. Recently, an increasing interest also has emerged for the synthesis of selenium-containing polymers due to their potential applicability as biomaterials.^18^,^19^

### Biological background

Peptide toxins form an important class of disulfide bond containing peptides and proteins. One particular group of peptide toxins isolated from scorpions and other venomous animals targets Kv1.3, the voltage-gated ion channel of human T lymphocytes.^20^ The Kv1.3 channel plays a crucial role in antigen-induced activation and proliferation of human T cells in general, and in those of effector memory T cells in particular.^21^ These latter cells are responsible for tissue damage in certain autoimmune diseases such as multiple sclerosis, type I diabetes, and rheumatoid arthritis.^22^ High affinity and selectivity Kv1.3 inhibitors persistently inhibit the proliferation of effector memory T cells thereby ameliorating the symptoms of the disease in animal models. This indicates the great therapeutic value of the Kv1.3 inhibitors.^22^–^24^

Anuroctoxin (AnTx), a 35-amino-acid scorpion toxin having four disulfide bridges, is a high affinity blocker of Kv1.3 channel, but also blocks Kv1.2.^25^ Improving selectivity of the toxin for Kv1.3 over Kv1.2 while keeping the high affinity for Kv1.3 opens the door to the toxin's therapeutic use. In line with these, we have constructed a double substituted analog of AnTx, [N17A/F32T]-AnTx, which is characterized by comparable Kv1.3 affinity to the wild-type peptide but shows a 2500-fold increase in the selectivity for Kv1.3 over Kv1.2 and other ion channels tested (Kv1.1 and KCa3.1).^26^ Thus, [N17A/F32T]-AnTx may serve as an excellent template for a novel therapeutically applicable peptide, and, interestingly, understanding the structural features of this peptide may lead to a better understanding of the nature of the selectivity of scorpion toxins between Kv1.3 and Kv1.2.

In the present work we report the chemical synthesis of a Sec-analog of [N17A/F32T]-AnTx during which – for the first time to our knowledge – the replacement of all four disulfide bonds with isosteric diselenide bonds has been achieved. Using a combined NOE- and ^77^Se NMR-based approach unequivocal assignment of the diselenide (disulfide)-bond connectivities was also accomplished. Moreover, the significant broadening of some of the ^77^Se resonances revealed the presence of slow conformational exchange processes which have been subsequently interpreted by molecular dynamics (MD) simulations.

## Results and discussion

### Synthesis of the Sec-analog of [N17A/F32T]-AnTx mutant

**Scheme 1:**
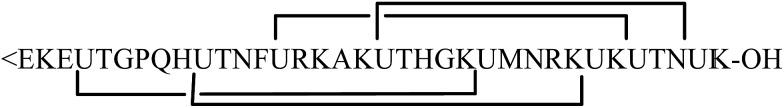
Amino acid sequence of Sec-[N17A/F32T]-AnTx with four diselenide bonds.

For the preparation of the anuroctoxin derived peptides, both the Boc and Fmoc chemistry were used previously with success.^26^ Since the stability of several Boc compatible Se protecting groups is significantly lower than the corresponding sulfur protecting groups (*e.g.*, BnSe^–^ is a much better leaving group than the corresponding sulfur derivative^12^,^27^), for the solid-phase synthesis of selenocysteine analog of [N17A/F32T]-AnTx mutant, the Fmoc/^*t*^Bu strategy was applied. Correspondingly, the related N^α^-Fmoc derivative of the 4-methoxy-benzyl protected selenocysteine^8^ was synthesized and used as replacement for all eight cysteines in the original sequence. For the linear Sec(Mob) protected [N17A/F32T]-AnTx analog, a standard synthetic protocol was applied with DCC/HOBt (1 : 1) as coupling agent in a 3-fold molar excess. The removal of the Mob group from the selenocysteine residues can be carried out, according to previous studies, under various conditions, *e.g.*, acidic treatment, I_2_, or applying a sub-stoichiometric amount of 2,2′-dithiobis(5-nitropyridine). With regard to the complexity of the amino acid sequence of the peptide, we decided to achieve the cleavage from the resin together with the protecting groups commonly used in Fmoc/^*t*^Bu synthesis and removal of the Mob group from the selenocysteine side-chains in a two-step protocol. Acidolytic cleavage of the Sec(Mob) peptide from the resin was performed under various conditions (TFA/H_2_O/TIS cocktail in 93/5/2, v/v, at low temperature for 2 h; TFA/H_2_O/TIS cocktail in 93/5/2, v/v at room temperature for 2.5 h) and in both cases the crude product obtained consisted mainly of the compound with all eight Mob groups intact besides compounds which had partially removed one, two, or even three Mob groups. In order to avoid incomplete removal of the side-chain protecting groups, and considering the fact that it follows Mob group de-protection, we decided to carry on with this complex reaction mixture. For Mob de-protection/cyclization we tried procedures well established in cysteine chemistry,^27^,^28^ like the DMSO/TFA (v/v) procedure (10% DMSO/TFA and 4% DMSO/TFA) and the I_2_-mediated method (added quantitatively or in excess), but in all cases we obtained a very complex mixture of compounds from which the desired diselenide-bridged peptide could not be isolated. Cleavage of the *p*-methoxybenzyl group and intramolecular oxidation to the diselenide peptide was successfully achieved with the more gentle 2,2′-dithiobis(5-nitropyridine) (DTNP) in TFA. Incubation of the protected Sec-containing peptide in TFA with DTNP resulted in effective protecting group removal and its substitution by the 2-(5-nitropyridyl) (Npys) group derived from DTNP fragmentation, independent of the number of DTNP equivalents and peptide concentration used. After test reactions, a final 4 mM peptide concentration in TFA was used with 1 equiv. DTNP incubation for 1 h. The reaction was quenched *via* precipitation into cold diethyl ether and, after isolation of the precipitate, the crude material was treated with one equivalent of l-cysteine in pH 8 NH_4_OAc buffer solution in order to induce collapse into the desired multiple diselenide-peptide. HPLC analysis of the resulting solution showed an almost instantaneous transformation of the hemi-reduced intermediate into the multiple diselenide compound (Scheme 1).

The HPLC trace of the folded, purified tetra diselenide toxin analog is shown on Fig. S1.† The spectra of mass spectrometric analyses of the multiple selenocysteine peptide recorded with quadrupole ESI and Q-TOF spectrometers are depicted in Fig. S2–S5.† The characteristic isotopic selenium abundance (Fig. S5†) is more complicated than the sulfur containing ones, but a careful study led to the justification of the correct molecular ion (Fig. S4†). Subsequent NMR studies and biological measurements of the isolated main product proved that all four diselenide bridges are completely analogous with the disulfide connectivities from the native [N17A/F32T]-AnTx peptide.

### The Sec-analog of [N17A/F32T]-AnTx retains its biological activity

**Fig. 1:**
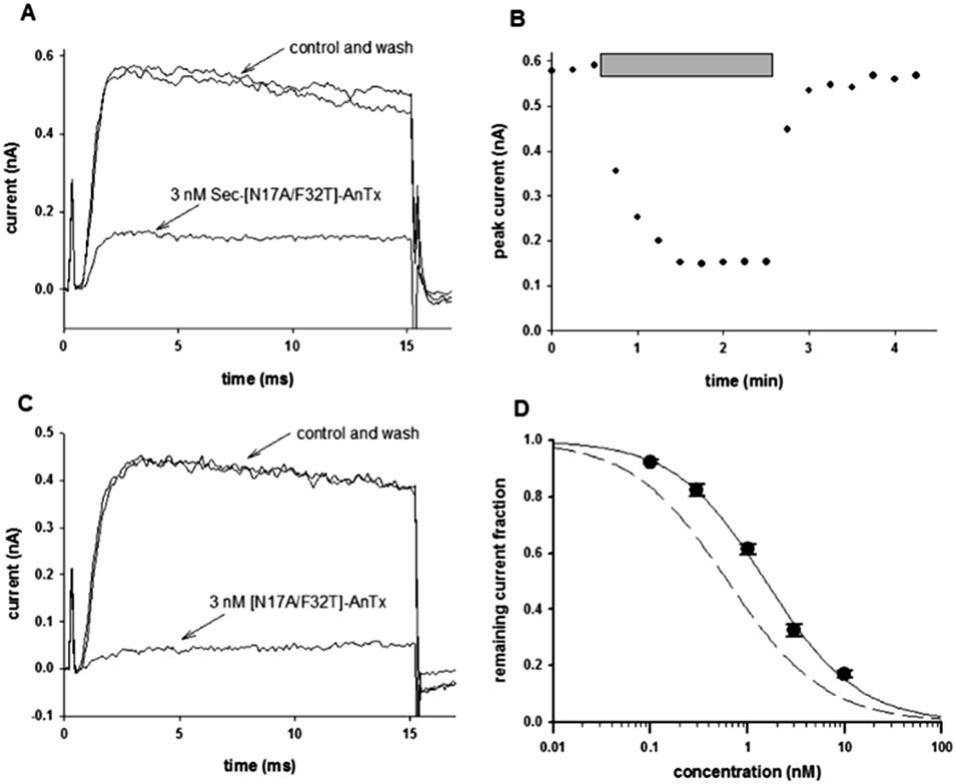
Effect of AnTx variants on Kv1.3 channel. Whole-cell Kv1.3 current traces were measured in activated human lymphocytes in whole cell configuration. Cells were held at –100 mV then depolarized to +50 mV for 15 ms every 15 s. The bath was perfused continuously. (A), The traces show the K^+^ current before the application of the toxin (control), after the equilibration of the block in the presence of 3 nM Sec-[N17A/F32T]-AnTx (as indicated) and after recovery from block during the perfusion of the bath with toxin-free solution (wash). (B), The blocking effect was fully reversible. Peak K^+^ currents were determined and plotted as a function of time. Grey bars mark the presence of 3 nM Sec-[N17A/F32T]-AnTx in the bath solution. Other experimental details are as in A. (C), The traces show the K^+^ current before the application of the toxin (control), after the equilibration of the block in the presence of 3 nM [N17A/F32T]-AnTx (as indicated) and after recovery from block during the perfusion of the bath with toxin-free solution (wash). (D), Concentration-dependence of K^+^ current block by Sec-[N17A/F32T]-AnTx. The remaining fraction of the Kv1.3 current was calculated as *I*/*I*_0_, where *I*_0_ and *I* are the peak K^+^ currents measured in the control solution and upon reaching equilibrium block during bath perfusion with the test solution containing the toxin at indicated concentrations, respectively. The voltage protocol and other experimental conditions were the same as in (A). The superimposed solid line is the Hill equations fitted to the data points (see Experimental). The best fit yielded IC_50_ = 1.54 nM, and H = 0.93. Error bars indicate SEM (*n* = 3–5). The dashed line shows the dose–response relationship of [N17A/F32T]-AnTx which is characterized by IC_50_ = 0.63 nM and H = 0.88 (data from ref. 26).

Prior to obtaining detailed structural information one key question remained to be answered, namely, whether the Sec-analog of [N17A/F32T]-AnTx still blocks the Kv1.3 channel with high affinity. To this end, the Sec-analog was tested on Kv1.3 expressed in human peripheral blood lymphocytes. Fig. 1 shows that the peptide in 3 nM concentration blocks approx. 75% of the peak current (Fig. 1A), which is comparable to the inhibition of Kv1.3 with the double mutated peptide [N17A/F32T]-AnTx at identical concentration (89% inhibition in the latter case,^26^Fig. 1C). Fig. 1B shows that the block of Kv1.3 develops quickly and it is rapidly reversible by perfusing the bath with toxin-free solution. The dose–response relationship obtained for Kv1.3 inhibition by Sec-[N17A/F32T]-AnTx (Fig. 1D) indicates an IC_50_ of 1.54 nM, which is comparable to that of [N17A/F32T]-AnTx (IC_50_ = 0.63 nM, dashed line). The Hill coefficient was close to 1 for both peptides (H = 0.93 for Sec-[N17A/F32T]-AnTx and H = 0.88 for [N17A/F32T]-AnTx) which argues that the peptide:channel stoichiometry of inhibition is 1 : 1, as predicted by currently accepted models of toxin binding to the channels.^29^

In summary, the key pharmacological property of [N17A/F32T]-AnTx, *i.e.*, the high affinity block of the Kv1.3 current, is retained in Sec-[N17A/F32T]-AnTx so the peptide is functionally active.

### NMR investigation of the Sec-analog of [N17A/F32T]-AnTx

**Fig. 2:**
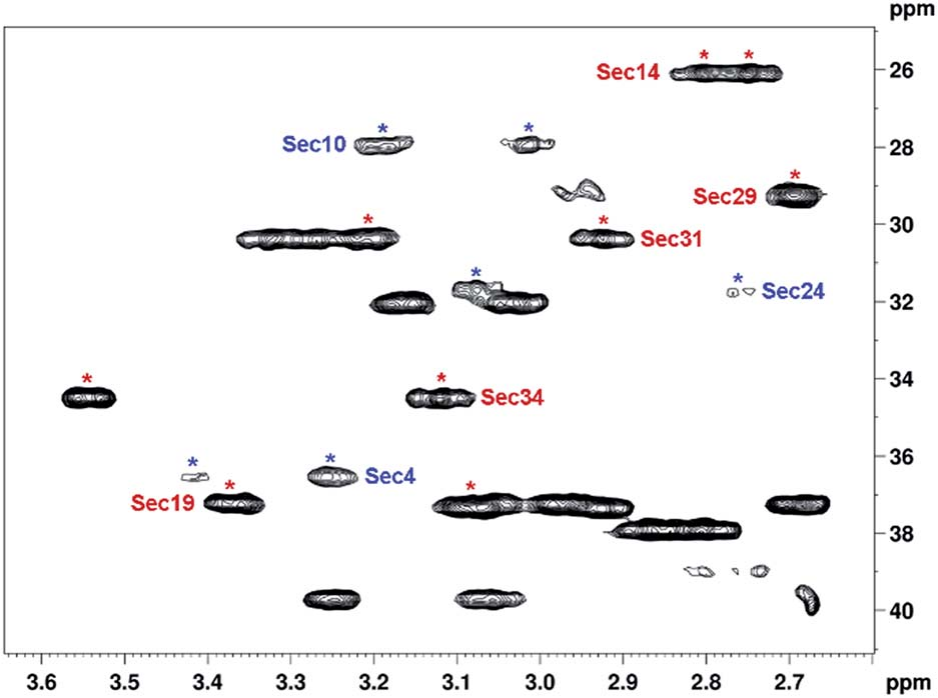
The H_β_/C_β_ region of ^1^H-^13^C HSQC spectrum of Sec-[N17A/F32T]-AnTx. Broadened (lower intensity) H_β_–C_β_ cross peaks of Sec4, Sec10 and Sec24 residues are indicated by blue asterisks. H_β_–C_β_ cross peaks of the remaining Sec residues are labelled by red asterisks.

Almost complete assignments of ^1^H, ^13^C and ^15^N resonances of Sec-mutant have been achieved using standard solution state NMR techniques. The ^1^H resonances were assigned based on two-dimensional (2D) homonuclear correlation spectra including ^1^H-^1^H COSY, TOCSY and NOESY. Whereas the well-resolved cross peaks of ^1^H-^13^C and ^1^H-^15^N HSQC spectra allowed the assignment of ^13^C and ^15^N resonances (see Table S1 in the ESI†), it should be mentioned that some of the H_β_–C_β_ cross peaks, such as of Sec4, Sec10 and Sec24 residues, are significantly broadened and have, therefore, lower intensities in the ^1^H-^13^C HSQC correlation map (Fig. 2); this is indicative of slow conformational dynamics occurring in the vicinity of those residues. Note that similar exchange mediated line-broadening was observed in the ^1^H-^15^N HSQC spectrum for T5, Q8, H9, U10, T11 and K30 residues (data not shown). It was also found that the Sec-derivative possesses almost identical ^1^H, ^13^C, and ^15^N chemical shifts as the S-containing peptide (selected regions of ^1^H-^13^C HSQC spectra of the two peptide analogs are shown in Fig. S6†), suggesting high similarity of their three-dimensional structures.

The oxidized state of all eight Sec residues incorporated into the peptide could be unequivocally proved on the basis of the characteristic H_β_/C_β_ and Se chemical shifts of the Sec residues (Table S1†). The MS data also supported the formation of four intramolecular diselenide bonds.

**Fig. 3:**
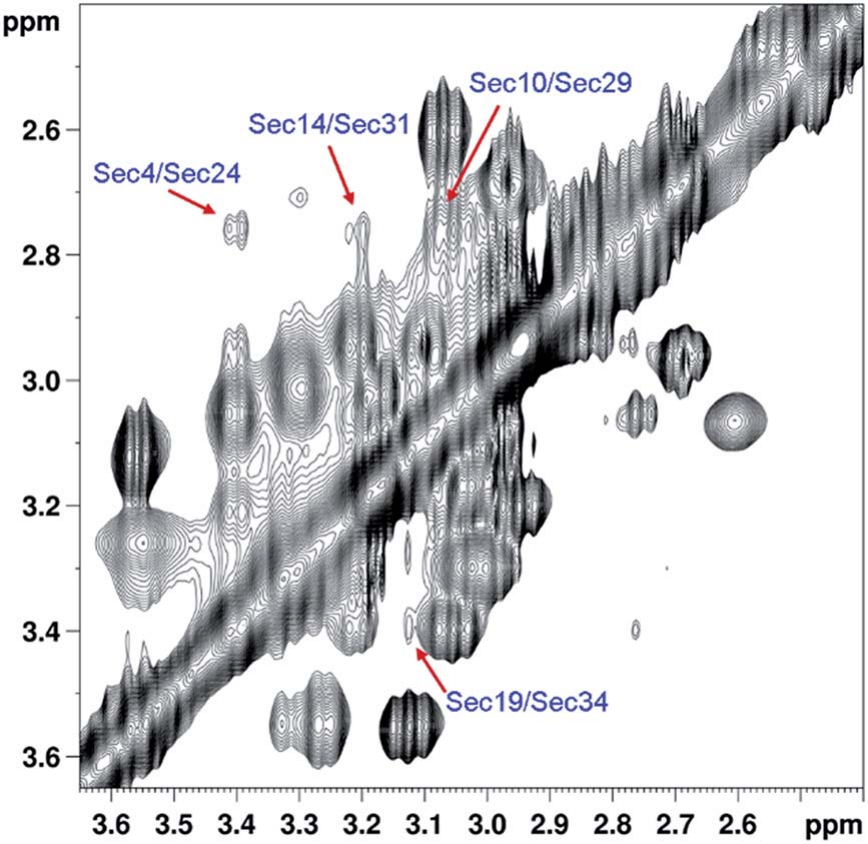
Partial contour plot of ^1^H-^1^H NOESY spectrum (500 MHz, *τ*_m_ = 300 ms) of Sec-[N17A/F32T]-AnTx. NOE cross peaks between the β-methylene protons of covalently linked Sec residues are indicated by arrows.

To establish the location of bridged residues, ^1^H-^1^H 2D NOESY spectra were recorded with 150 ms and 300 ms mixing. However, even using longer mixing time (300 ms), NOE cross peaks between the β-methylene protons of the covalently linked Sec residues could be hardly detected. This may be due partly to the strong intraresidue cross-relaxation between the geminal β-protons of Sec, partly to the line broadening caused by slow conformational dynamics around the Se–Se bridge, and also to the severe signal overlap in the region of interest. Specifically, inspection of the NOESY spectrum shown in Fig. 3 reveals only one Se–Se bond unequivocally, which can be assigned to the Sec4–Sec24 bridge. Unambiguous assignment of the other inter-Sec(H_β_) cross peaks is hampered by the strong signal overlap.

To take advantage of our ‘Se NMR-spies’ incorporated into the peptide sequence, multiple bond ^1^H-^77^Se correlation spectra were recorded using standard HMQC or HMBC experiments. Sites around disulfide/diselenide bridges in peptides/proteins, however, are often regions of conformational exchange. The exchange process, in general, can be detrimental to the quality of NMR spectra, resulting in broad and very weak, or even unobservable signals, and which may be detrimental to structure elucidation and, in particular, to the NMR-based characterization and assignment of disulfide/diselenide bonds. To our disappointment, even after several attempts using a different experimental setup (see ESI† for details and selected examples), only a few and exclusively intraresidue ^1^H-^77^Se correlations could be observed, probably due to conformational exchange line broadening.

To improve the sensitivity of ^1^H-^77^Se multiple bond correlation measurements, a modified, ^77^Se-decoupled variant of the CPMG-HSQMBC experiment^30^–^32^ has been developed in our group. This experiment considerably reduces the adverse line broadening effect of conformational exchange and also efficiently suppresses the undesired co-evolution of proton–proton couplings (such as the large geminal coupling between the H_β_ protons of Sec) during the long scalar coupling evolution period matched to the intra- and interresidue multiple bond ^1^H-^77^Se couplings. To minimize heating of the probe electronics and the sample during the long duration of CPMG pulse train, low-power composite 180° pulses were applied on both the proton and Se nuclei. Note that, similarly, CPMG pulse trains have been successfully employed in HSQC experiments to reduce the undesired effect of exchange processes and, as a consequence, for better observation of exchange broadened signals in nucleic acids and proteins.^33^–^35^

**Fig. 4:**
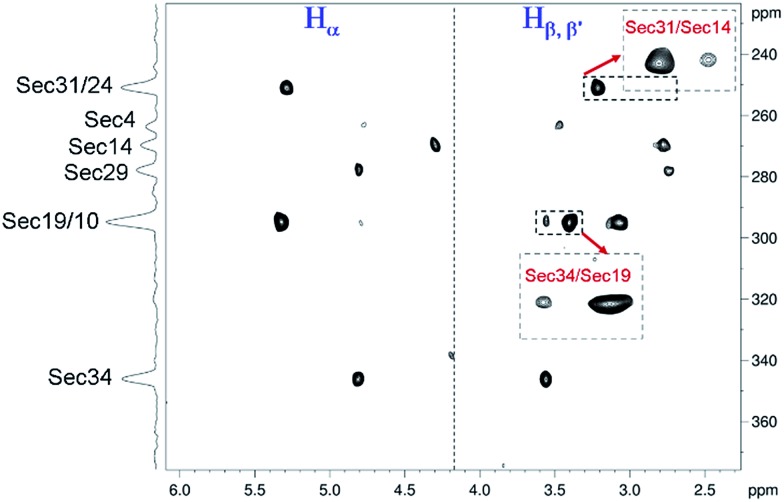
^1^H-^77^Se multiple bond correlation spectrum of Sec-[N17A/F32T]-AnTx collected with a refocused ^77^Se-decoupled CPMG-HSQMBC experiment at 318 K and natural abundance of ^77^Se isotope. Heteronuclear coupling evolution time was set to 33 ms. Assignment of ^77^Se resonances are given on the projection at left side. Interresidue ^1^H-^77^Se cross peaks detected are shown in insets at lower intensity threshold and provide direct evidence of Se–Se bridges between Sec14–Sec31 and Sec19–Sec34 residues, respectively.

The details of the experiment proposed here (scheme in Fig. S7† and Bruker code of the pulse sequence are given in ESI†) and comparison of its performance to the classical HMQC/HMBC methods are demonstrated in the ESI (Fig. S8–S10†). Recording several ^1^H-^77^Se CPMG-HSQMBC spectra with the sequence of Fig. S7† at different temperatures and using evolution times matched to different long-range couplings, we found that the largest number of connectivities could be detected at 318 K with the coupling evolution time set to 30–35 ms. The resulting spectrum shown in Fig. 4 allowed unambiguous assignment of all eight Se-resonances (as given on the projection at left), though some of the correlations are weak due to exchange broadening as noticed. Nevertheless, the cross peaks enlarged and shown in insets at lower intensity threshold could be assigned to provide direct evidence of two additional Se–Se pairings, between Sec14–Sec31 and Sec19–Sec34 residues, respectively.

Since the chemical shifts (C_β_, Se) of Sec-s already confirmed that all eight Sec residues are involved in diselenide bonds, thus the remaining, unassigned Se–Se connectivity could be unambiguously assigned to the Sec10 and Sec29 residues.

### Molecular dynamics (MD) simulation

**Fig. 5:**
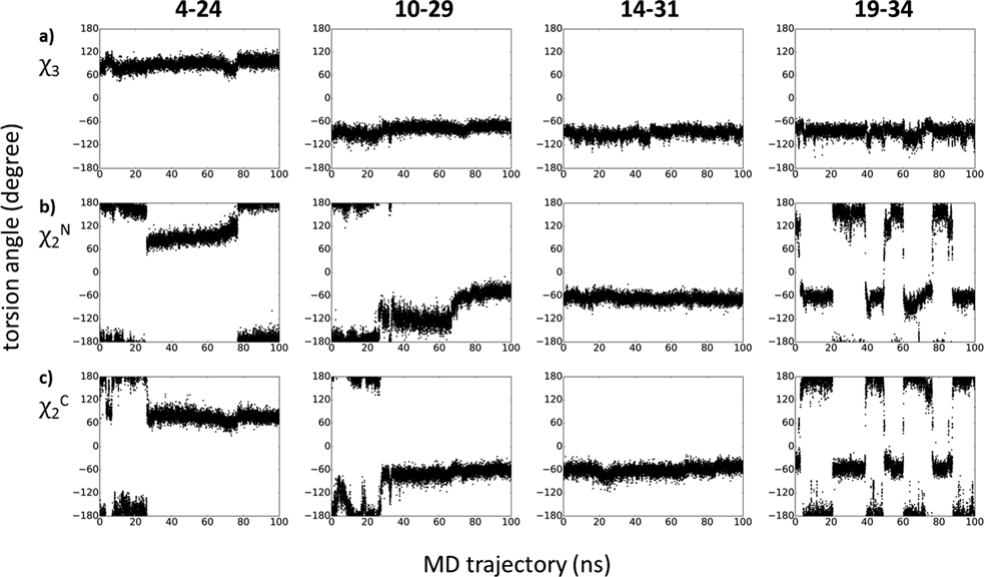
Development of torsion angles along the trajectory for disulfide bridges 4–24, 10-29, 14–31 and 19–34. The first row (a) shows the *χ*_3_ angle is the torsion angle around the disulfide bond (as defined by atoms CB^N^-SG^N^-SG^C^-CB^C^). The *χ*_2_ sidechain torsion angle is shown for (b) the N terminal (as defined by atoms CA^N^-CB^N^-SG^N^-SG^C^) and (c) the C terminal residue (as defined by atoms SG^N^-SG^C^-CB^C^-CA^C^) of the cysteine pair in the bridge.

MD calculations were found to be useful tools to analyze disulfide bridge connectivities in small disulfide-rich proteins.^36^,^37^ In order to find an explanation for the observed NMR behaviour of peptide sites around diselenide bridges, we carried out a 100 ns molecular dynamics (MD) simulation on the [N17A/F32T]-AnTx peptide. Since replacement of disulfide bridges with diselenide bonds has been demonstrated to be bioisoteric due to high similarity between the conformational properties of sulfur and selenium,^11^ the simulations were carried out on the disulfide bridge containing peptide. The *χ*_3_ angle has taken up conformations around the canonical ±90° and has mostly persisted in the conformation presented in the starting structure shown in Fig. 5a. The dynamic behavior of the different S–S bridges in the trajectory could be classified into three groups according to the conformation along the side chain *χ*_2_ dihedral angles of the cysteine residues (Fig. 5b and c): the conformation around the 14–31 bridge was static apart from small fluctuations, the 19–34 bridge was most dynamic with frequent transitions between different states, and the bridges 10–29 and 4–24 showed intermediate dynamic behavior with less frequent or a single transition between conformations. Thus, the strong intra/inter-residue HSQMBC correlations for ^77^Se resonances in bridge 14–31 are due to the lack of any exchange process for the nuclei involved, while the nuclei in bridge 19–34 may experience fast exchange line narrowing. The weaker correlations for the connectivities in bridges 10–29 and 4–24 could be attributed to the presence of intermediate conformational exchange broadening.

## Conclusions

In summary, a Sec-analog of the biologically active [N17A/F32T]-AnTx peptide toxin containing four diselenide bonds has been produced with chemical synthesis. To the best of our knowledge, this is the first time all disulfide bonds in a protein were replaced by chemical synthesis with isosteric diselenides. We found that the Sec-derivative displayed similar voltage-gated potassium channel (Kv1.3) blocking potency and almost identical ^1^H, ^13^C and ^15^N chemical shifts as the S-containing peptide, suggesting high similarity of their three-dimensional structures including their Se–Se/S–S bridge pattern, respectively.

Using a combined NMR approach relying on characteristic chemical shifts, ^1^H-^1^H NOEs and ^1^H-^77^Se multiple bond connectivities, the diselenide network of the Sec-analog was unequivocally disclosed. To improve the sensitivity of ^1^H-^77^Se correlation NMR experiment for peptides with multiple diselenide bonds and in the presence of exchange broadening, a modified CPMG-HSQMBC pulse sequence was designed and applied. The CPMG-cycles employed efficiently reduce the loss of spin coherence due to both chemical exchange and undesired evolution of proton–proton couplings.

Finally, NMR experiments complemented with MD simulations allowed us to explore and characterize the conformational dynamics of diselenide/disulfide bonds.

## Experimental

### Electrophysiology

#### Cells

Kv1.3 currents were measured on human peripheral lymphocytes obtained from healthy volunteers. Mononuclear cells were isolated using the Ficoll-Hypaque density gradient separation technique and cultured in 24-well culture plates at 37 °C in a 5% CO_2_ incubator in RPMI 1640 medium supplemented with 10% fetal calf serum (Sigma-Aldrich), 100 μg mL^–1^ penicillin, 100 μg mL^–1^ streptomycin, and 2 mM l-glutamine (density, 5 × 10^5^ cells per mL) for 2 to 5 days. To increase K^+^ channel expression 5, 7.5, or 10 μg mL^–1^ phytohemagglutinin A (Sigma-Aldrich) was added to the medium.

#### Electrophysiology

Electrophysiological recordings were carried out with the patch-clamp technique in the voltage-clamp mode in whole cell configuration. For the recordings an Axon Axopatch 200B amplifier and Axon Digidata 1440 digital interface were used (Molecular Devices, Sunnyvale, CA). Micropipettes were pulled from GC 150 F-15 borosilicate capillaries (Harvard Apparatus Kent, UK) resulting in 3 to 5 MΩ resistance in the bath solution, which consisted of 145 mM NaCl, 5 mM KCl, 1 mM MgCl_2_, 2.5 mM CaCl_2_, 5.5 mM glucose, 10 mM HEPES, pH 7.35, 302–308 mOsM L^–1^. The pipette filling solution contained 140 mM KF, 2 mM MgCl_2_, 1 mM CaCl_2_, 10 mM HEPES and 11 mM EGTA, pH 7.22, 295 mOsM L^–1^.

Kv1.3 currents were elicited by 15 ms long depolarization impulses to +50 mV from a holding potential of –100 mV every 15 s. To acquire and analyze the measured data a pClamp10 software package was used. Current traces were lowpass-filtered by the analog four-pole Bessel filters of the amplifiers. The effect of the toxin in a given concentration was determined as remaining current fraction (RF = *I*/*I*_0_, where *I*_0_ is the peak current in the absence of the toxin and I is the peak current at equilibrium block at a given toxin concentration). Points on the dose–response curves represent the mean of 5 independent measurements where the error bars represent the S.E.M. Data points were fitted with a two-parameter Hill equation, RF = IC_50_^H^/(IC_50_^H^ + [Tx]^H^), where IC_50_ is the dissociation constant, H is the Hill coefficient and [Tx] is the toxin concentration.

### NMR spectroscopy

3.9 mg peptide toxin was dissolved in 280 μL buffer (90 : 10 H_2_O/D_2_O, 10 mM Na_3_PO_4_, pH 5.7, 50 mM NaCl, 1 mM NaN_3_) for all NMR runs whereas 7.9 mg was disssolved in D_2_O buffer for ^77^Se NMR measurements and transferred into a Shigemi NMR tube. All NMR spectra were recorded on an Avance II 500 (Bruker, Rheinstetten, Germany) spectrometer at 303 K, if not indicated otherwise. ^1^H chemical shifts are referenced to sodium 2,2-dimethyl-2-silapentane-5-sulfonate (0 ppm), while heteronuclear shifts are referenced indirectly based on the gyromagnetic ratios for ^13^C, ^15^N, and ^77^Se, respectively. All heteronuclear correlation spectra (^1^H-^13^C and ^1^H-^15^N HSQC, selenium-decoupled ^1^H-^77^Se CPMG-HSQMBC, HMBC and HMQC) were acquired at natural abundance. The ^1^H-^77^Se multiple bond correlation spectra were recorded at 283, 303, 313, and 318 K, respectively using scalar coupling evolution time matched to 10, 12, 15, 17, 20, and 30 Hz, typically in overnight experiments. 2D ^1^H-^1^H NOESY spectra using a Watergate sequence for water suppression were recorded with 150 and 300 ms mixing, respectively. TOCSY mixing was set to 80 ms. The ^1^H, ^13^C, ^15^N, and ^77^Se 90° hard pulses were of 11.5, 13.0, 29.0, and 15.0 μs duration, respectively.

### Computational methods

All MD simulations were performed using AMBER version 12.^38^ The AMBER ff99SB force field^39^ for the peptides and the TIP3P model^40^ for water were used. The cut-off used for non-bonded interactions was 8 Å. The particle-mesh Ewald^41^ procedure was used to describe long-range electrostatic interactions with a maximal grid spacing of 1 Å. Periodic boundary conditions were applied with a truncated octahedron geometry. The SHAKE^42^ algorithm was used to keep the bond lengths of hydrogen atoms rigid allowing a timestep of 2 fs to be used.

Since there is no selenium atom type implemented in the chosen force field, we used sulfur in place of selenium. Comparison of the properties of sulfur and selenium shows that the structure and dynamics of the selenium-containing peptide should be very similar to the sulfur-containing one. This assumption was confirmed by comparison of the ^1^H, ^13^C, and ^15^N chemical shifts, which were strongly correlated.

#### Protocol

First minimization in 10 000 steps was performed to remove bad contacts switching from steepest descent to a conjugate gradient algorithm after 100 steps. After minimization at a constant pressure MD was carried out to equilibrate the system for 0.5 ns, during which the density stabilized at around 0.9965 g cm^–3^, and the temperature settled at *ca.* 300 K. Subsequently, a classical constant total energy MD producing micro canonical NVE ensemble was carried out for 100 ns.

All simulations were run in a single Nvidia GTX680 GPU using the GPU^43^,^44^ implementation of the pmemd provided in the AMBER12 simulation package. Using the described protocols a 100 ns-long calculation of the peptide took about 25 h to complete.

#### Analysis

Translational centre of mass motions were removed every 1000 steps for NVE simulations. 50 000 coordinate snapshots were saved for analysis in all cases. The trajectories were analyzed with ptraj^45^ and visualized in VMD.^46^ The stability of the trajectories were analyzed using the mdout_analyser.py.

## Supplementary Material

Supplementary information
