# Supplementary material for: Probing pattern and dynamics of disulfide bridges using synthesis and NMR of an ion channel blocker peptide toxin with multiple diselenide bonds
Source: Chem Sci. 2015 Dec 21;7(4):2666–73. doi: 10.1039/c5sc03995a (PMC5477041; doi:10.1039/c5sc03995a)
Supplement: Supplementary file 1 [file SC-007-C5SC03995A-s001.pdf]

Electronic Supplementary Material (ESI) for Chemical Science.
This journal is © The Royal Society of Chemistry 2015

Electronic Supplementary Information

Probing pattern and dynamics of disulfide bridges using synthesis and NMR of an ion channel blocker peptide toxin with multiple diselenide bonds

Krisztina Fehér, István Timári, Kinga Rákosi, János Szolomájer, Tünde Zita Illyés, Ádám Bartók, Zoltán Varga, György Panyi, Gábor K. Tóth, Katalin E. Kövér

Synthesis of Sec-analog of [N17A/F32T]-AnTx mutant

Linear Sec-[N17A/F32T]-AnTx

GlpKEU(Mob)TGPQHU(Mob)TNFU(Mob)RKAKU(Mob)THGKU(Mob)MNRKU(Mob)KU(Mob)TNU(Mob)K-OH

M_w= 5338.69

N-9-fluorenylmethoxycarbonyl-Se-4-methoxybenzylselenocysteine [Fmoc-Sec-(Mob)-OH] was prepared from selenocystine in two steps according to the literature.[8] Manual synthesis was carried out on Fmoc-Lys(Boc)-Wang resin (loading: 0,36 mmol/g) with standard N^{α}-Fmoc chemistry on a 0.2 molar scale. For Fmoc-deprotection 20% (v/v) piperidine in DMF (10 ml) was applied (5+15 min), a 3 fold molar excess of DCC-HOBt-activated N^{α}-Fmoc-amino acids (0.6-0.6 mmol) were coupled step by step. After completion of the coupling reaction, which was proven by negative ninhydrin test, the resin was washed with DMF, MeOH, and again with DMF (10-10 ml). Amino acids used for chain elongation were: Fmoc-Ala-OH (M_w = 329.36; 0.197 g, 0.6 mmol), Fmoc-Arg(Pbf)-OH (M_w = 648.8; 0.389 g, 0.6 mmol), Fmoc-Asn(Trt)-OH (M_w = 596.7; 0.358 g, 0.6 mmol), Fmoc-Gln(Trt)-OH (M_w = 610.7; 0.366 g, 0.6 mmol), Fmoc-Glu(O*t*Bu)-OH·H_2O (M_w = 443.5; 0.266 g, 0.6 mmol), Fmoc-Gly-OH (M_w = 297.3; 0.178 g, 0.6 mmol), Fmoc-His-OH (M_w = 619; 0.371 g, 0.6 mmol), Fmoc-Lys(Boc)-OH (M_w = 468.5; 0.281 g, 0.6 mmol), Fmoc-Met-OH (M_w = 371.5; 0.222 g, 0.6 mmol), Fmoc-Phe-OH (M_w = 387; 0.232 g, 0.6 mmol), Fmoc-Pro-OH (M_w = 337.4; 0.202 g, 0.6 mmol), Fmoc-*L*-Sec(Mob)-OH (M_w = 510.44; 0.306 g, 0.6 mmol), Fmoc-Thr(*t*Bu)-OH (M_w = 397.2; 0.238 g, 0.6 mmol), Glp-OH (M_w = 129.12; 0.077 g, 0.6 mmol). After completion of the chain elongation by subsequent coupling and deprotection, the peptide was cleaved from the resin with a mixture of TFA/water/TIS (93:5:2, v/v) at room temperature for 2.5 hours. The crude peptide was analyzed by RP-HPLC and Q-TOF mass spectrometer (M_w = 4369.89). The crude Se-protected Sec-[N17A/F32T]-AnTx peptide (100 mg) was dissolved in a small amount of TFA (5 ml) and 1 equiv. of 2,2'-dithiobis(5-nitropyridine) was added for each selenocysteine of the linear peptide (8 equiv., 0.046 g, 0.15 mmol; M_w = 310.31) and stirred at room temperature for 1 hour. At the end of the reaction time, cold diethyl ether was added to the reaction and the crude precipitated product was isolated using filtration. The crude isolate (96 mg) was dissolved in NH_4OAc buffer pH 8.5 (100 ml) and 1 equiv. of cysteine was added for each selenocysteine (8 equiv., ~ 20 mg, 0.176 mmol; M_w = 121.16) and stirred at room temperature for 15 min. After completion of the reaction, the solution was lyophilized. The resulted crude cyclic Sec-[N17A/F32T]-AnTx peptide was analysed and purified by RP-HPLC.

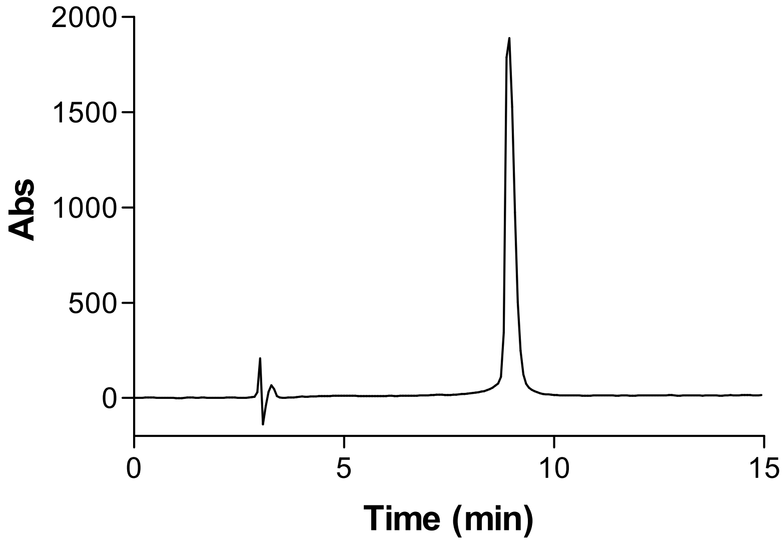

Figure S1 The HPLC profile of the purified octa Sec-analog of the [N17A/F32T]-AnTx mutant.

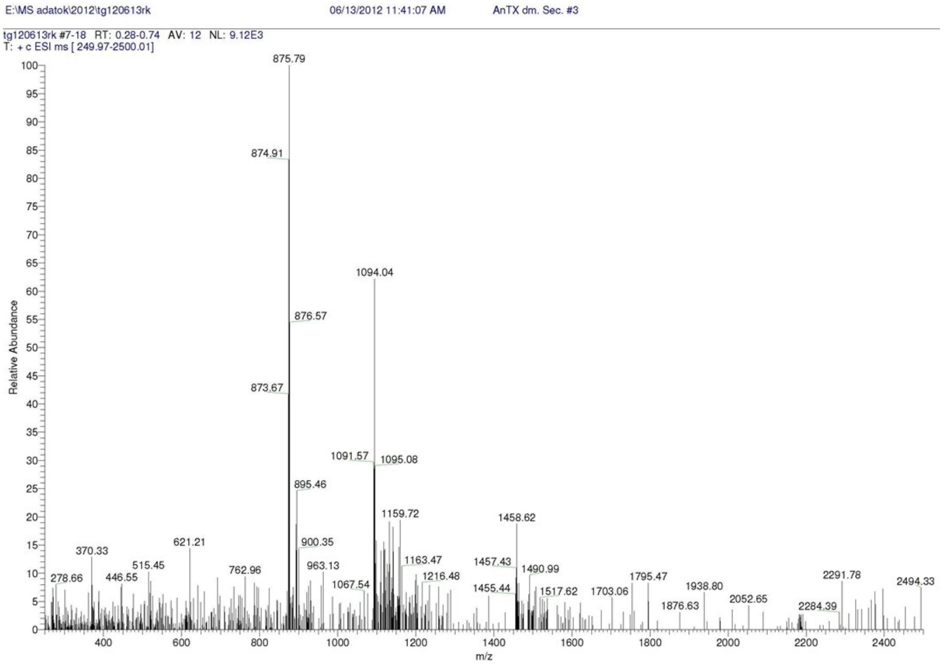

Figure S2 The quadrupole ESI MS spectrum of the purified octa Sec-analog of the [N17A/F32T]-AnTx mutant.

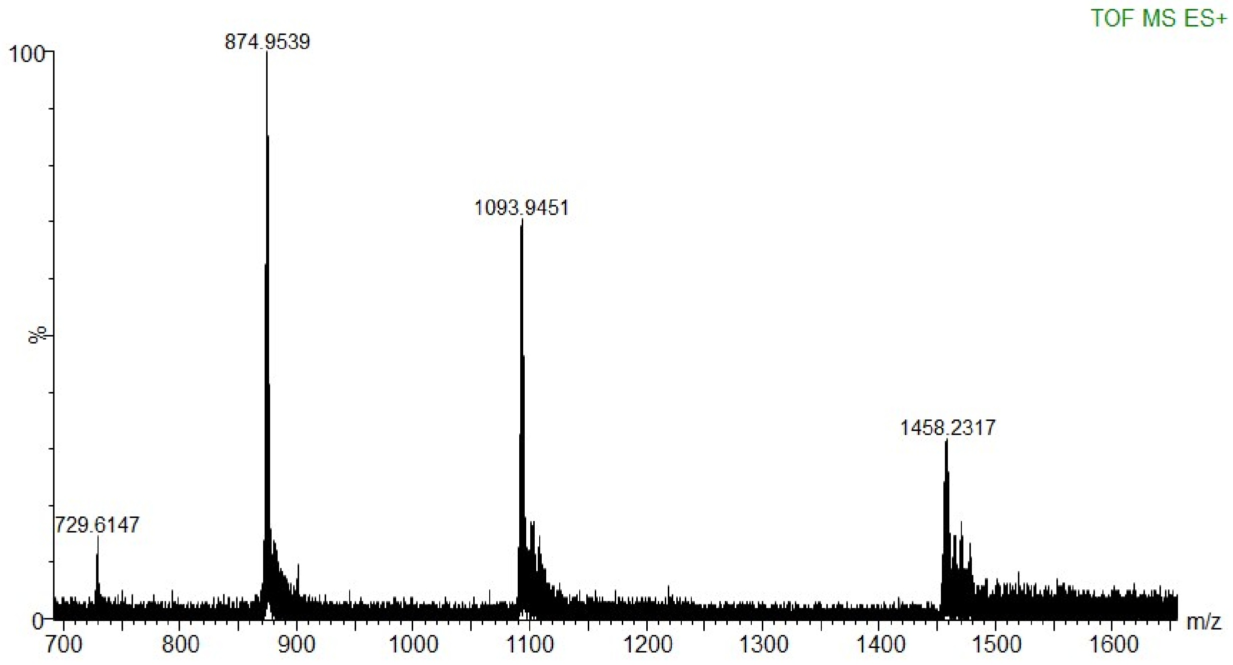

Figure S3 Q-TOF electrospray spectrum of the purified octa Sec-analog of the [N17A/F32T]-AnTx mutant.

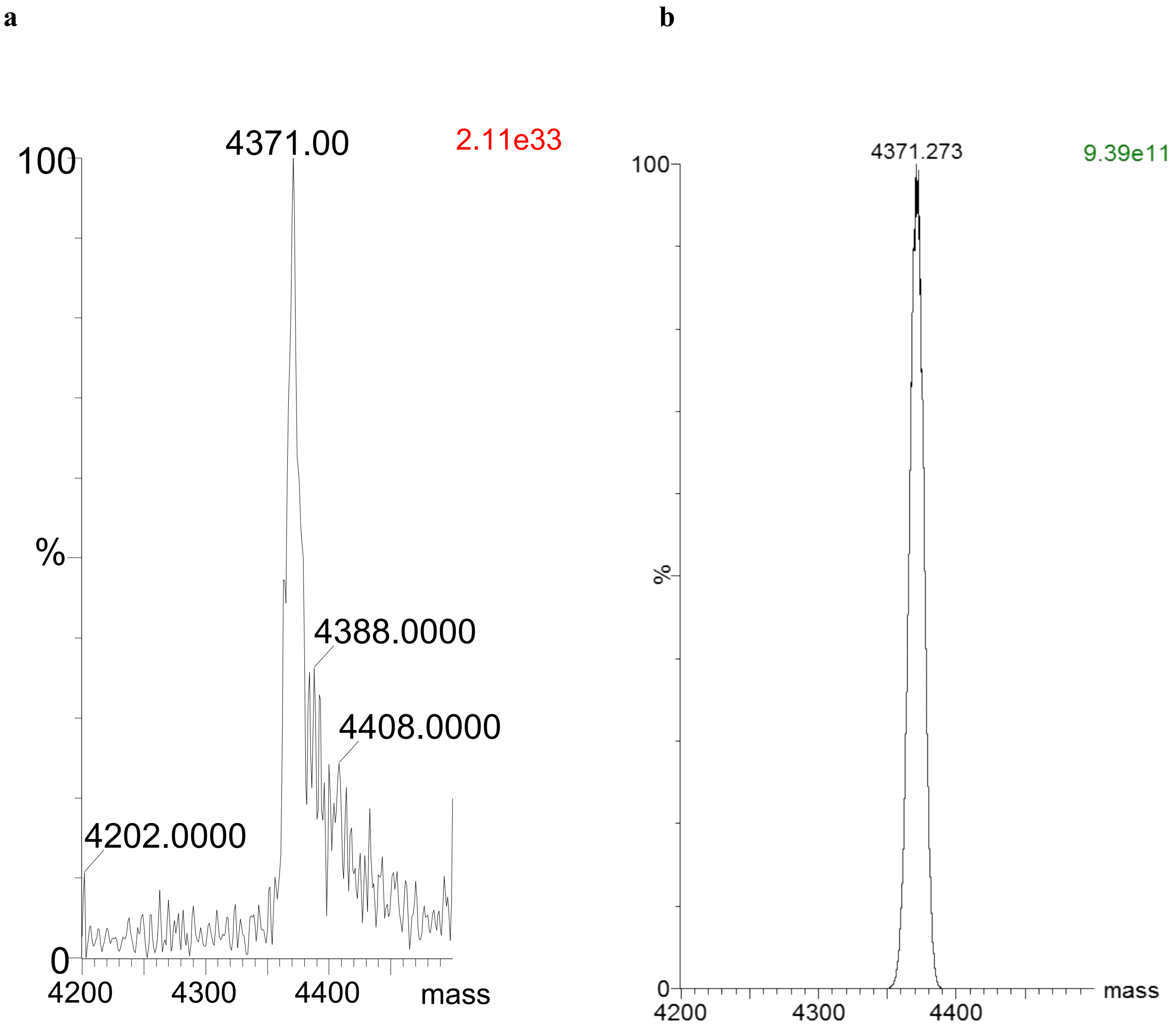

Figure S4 (**a**) Deconvoluted spectrum of the above (average mass) and (**b**) the calculated spectrum (average mass)

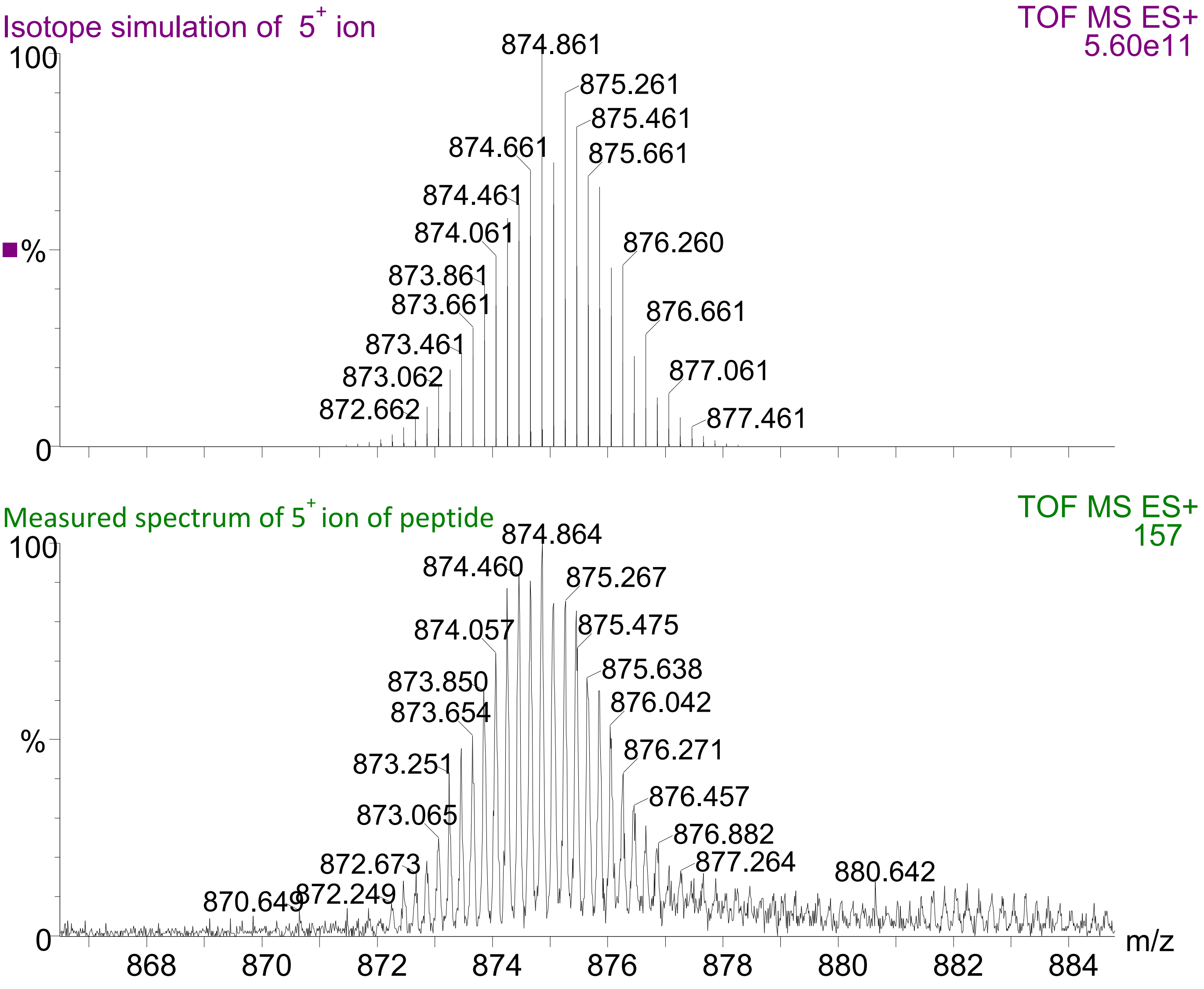

Figure S5 Characteristic isotopic selenium abundance seen in TOF MS spectrum of the purified octa Sec-analog of the [N17A/F32T]-AnTx mutant.

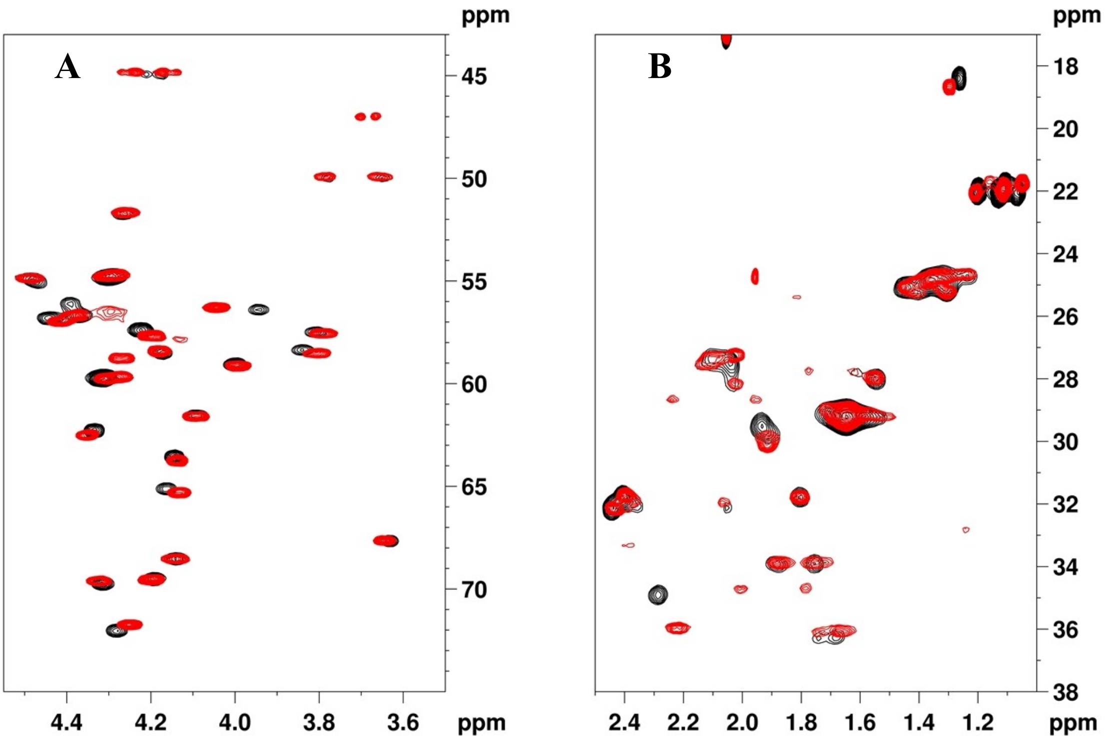

Figure S6 Overlay of ^{1}H-^{13}C HSQC spectra of the double mutant [N17A/F32T]-AnTx (black) and its Sec-analog (red). The H_{α}/C_{α} region (**A**) and the aliphatic side chain region (**B**) of the corresponding ^{1}H-^{13}C HSQC spectra are shown.

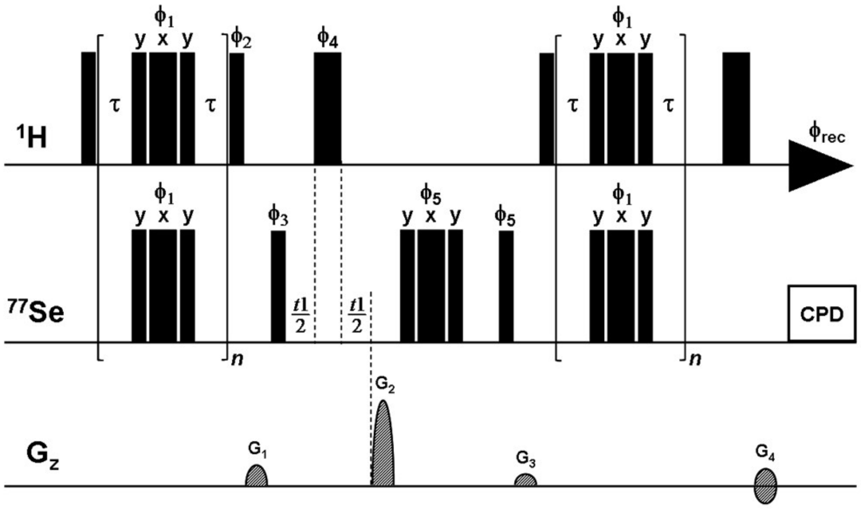

Figure S7 Pulse sequence scheme of the refocused ^{1}H-^{77}Se CPMG-HSQMBC experiment with ^{77}Se decoupling designed for the direct determination of diselenide-bond connectivities in case of exchange broadening. Narrow and wide filled bars correspond to 90° and 180° pulses respectively, with phase *x* unless indicated otherwise. ϕ_1 is incremented according to XY-16 cycles within the CPMG sequences, thus *n* should be adjusted to a multiple of 16. Other phases are $\phi_2 = y$; $\phi_3 = x, -x$; $\phi_4 = x, x, -x, -x$; $\phi_5 = x, x, x, x, -x, -x, -x, -x$; and $\phi_{rec} = x, -x, x, -x, -x, x, -x, x$. Delay τ was set to 150 μs. In order to provide simultaneous composite π pulses on the ^{1}H and the heteronucleus channel, power levels were carefully calibrated to give equal durations for proton and ^{77}Se pulses. Coherence order selection and echo-antiecho phase sensitive quadrature detection in the ^{77}Se dimension were achieved with gradient pulses G_2 and G_4 in the ratio 80 : 15.257. Purging gradient pulses G_1 and G_3 were set to 19% and 10% of maximum gradient strength (50 G/cm). Sine bell shaped gradient pulses of 1 ms duration were utilized, followed by a recovery delay of 200 μs. Waltz16 sequence was used for ^{77}Se decoupling during acquisition (CPD). Acquisition time (AQ) was carefully adjusted under 100 ms to minimize the heating of the sample.

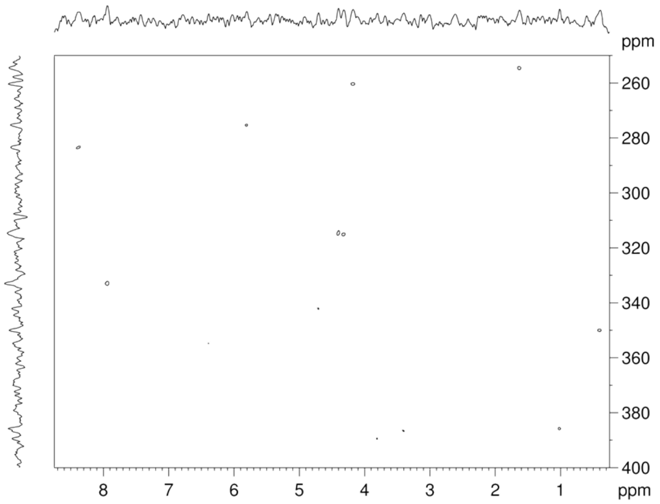

Figure S8 Conventional ^{1}H-^{77}Se HMQC spectrum of Sec-[N17A/F32T]-AnTx recorded with heteronuclear coupling evolution of 16.7 ms at 283 K and in experiment time of 32 h.
No ^{1}H-^{77}Se correlations could be detected.

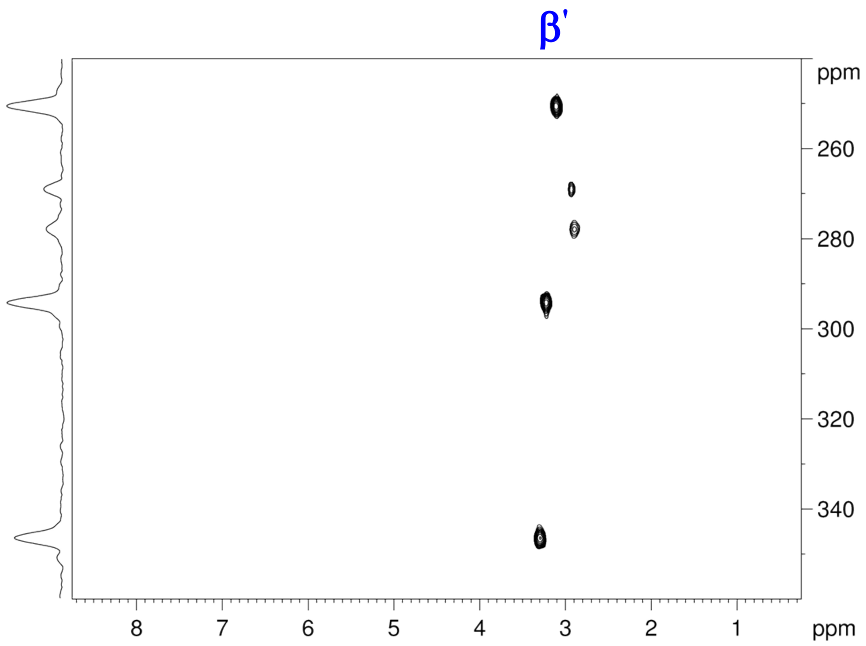

Figure S9 Conventional ^{1}H-^{77}Se HMQC spectrum of Sec-[N17A/F32T]-AnTx recorded with heteronuclear coupling evolution of 16.7 ms at 313 K and in experiment time of 40 h. Some intraresidue ^{1}H-^{77}Se multiple bond correlations are detectable.

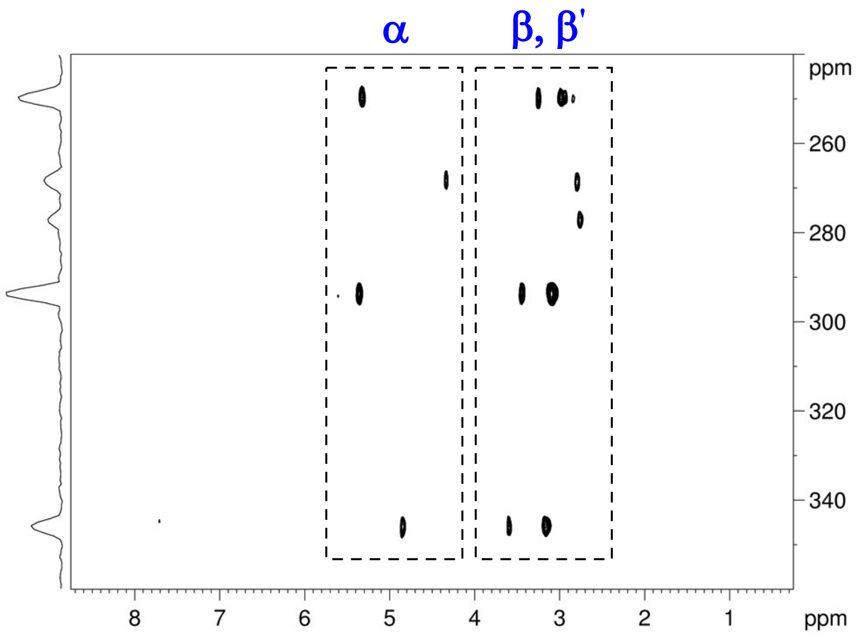

Figure S10 ^{1}H-^{77}Se CPMG-HSQMBC spectrum of Sec-[N17A/F32T]-AnTx recorded with heteronuclear coupling evolution of 16.7 ms at 313 K and in experiment time of 17 h. Almost all intraresidue ^{1}H-^{77}Se multiple bond correlations are observed.

Table S1 Assignment of ^{1}H, ^{13}C, ^{15}N and ^{77}Se resonances of Sec-[N17A/F32T]-AnTx peptide

Residue \ Atom	N	ND	NE	H	HA	HB	HG	HD	HE	HZ	CA	CB	CG	CD	CE	CZ	Se
**E1**	124.89	-	-	-	4.30	2.53, 2.53	2.43, 2.43	-	-	-	56.49	32.09	28.21	-	-	-	-
**K2**	-	-	-	7.64	4.03	-	-	-	-	-	56.26	-	-	-	-	-	-
**E3**	122.84	-	-	8.43	4.41	1.90, 1.90	2.21, 2.21	-	-	-	56.94	-	35.93	-	-	-	-
**U4**	-	-	-	7.84	4.73	3.25, 3.39	-	-	-	-	-	36.49	-	-	-	-	263.3
**T5**	-	-	-	8.85	4.33	4.32	1.10	-	-	-	62.46	69.58	21.92	-	-	-	-
**G6**	111.94	-	-	7.91	4.15, 4.23	-	-	-	-	-	44.80	0.00	-	-	- -	-	-
**P7**	-	-	-	-	4.12	2.36, 2.07	2.11	3.78, 3.65	-	-	63.75	-	-	49.90	-	-	-
**Q8**	120.43	-	112.07	9.10	4.17	2.01, 2.01	2.08, 2.38	-	6.88, 7.38	-	58.36	28.18	33.36	-	-	-	-
**H9**	118.35	-	-	7.81	4.63	3.25, 3.30	-	7.12	8.23	-	57.11	30.37	-	118.39	138.32	-	-
**U10**	118.00	-	-	8.11	4.72	3.18, 3.02	-	-	-	-	-	27.91	-	-	-	-	294.8
**T11**	118.04	-	-	7.92	3.63	4.13	1.20	-	-	-	67.63	68.51	22.03	-	-	-	-
**N12**	120.19	112.69	-	8.34	4.37	2.85, 2.79	-	6.96, 7.64	-	-	56.67	37.90	-	-	-	-	-
**F13**	122.24	-	-	8.35	4.08	3.05, 3.24	-	7.23	7.33	7.25	61.57	39.69	-	132.55	131.72, 130.3	2.00	-
**U14**	-	-	-	8.45	4.26	2.80, 2.75	-	-	-	-	59.62	26.07	-	-	-	-	269.9
**R15**	122.06	-	124.54	8.27	4.26	1.90, 1.92	1.77, 1.62	3.15, 3.15	7.12	-	58.73	30.02	27.73	-	-	-	-
**K16**	121.76	-	-	7.79	3.98	1.79, 1.79	1.41, 1.52	1.79, 1.62	3.13, 3.13	-	59.11	31.76	25.31	-	-	-	-
**A17**	121.27	-	-	7.04	4.25	1.29	-	-	-	-	51.65	18.68	-	-	-	-	-
**K18**	111.89	-	-	7.75	3.78	2.23, 1.95	1.31, 1.31	1.66, 1.63	2.94, 2.94	-	57.53	28.63	24.66	29.18	42.30	-	-
**U19**	120.03	-	-	8.12	5.31	3.36, 3.05	-	-	-	-	55.81	37.21	-	-	-	-	294.8
**T20**	111.71	-	-	7.96	4.13	4.19	1.11	-	-	-	65.27	69.50	21.94	-	-	-	-
**H21**	-	-	-	7.84	4.90	3.15, 3.02	-	7.14	8.45	-	54.73	32.04	-	120.07	136.71	-	-
**G22**	107.84	-	-	8.15	3.67, 5.08	-	-	-	-	-	46.90	8.00	-	-	- -	-	-
**K23**	120.79	-	-	8.60	4.48	1.73, 1.65	1.31, 1.31	-	2.91, 2.91	-	54.85	36.05	24.71	-	-	-	-
**U24**	125.49	-	-	8.99	4.94	3.06, 2.75	-	-	-	-	57.14	31.71	-	-	-	-	250.9
**M25**	128.05	-	-	8.86	4.68	1.99, 1.77	2.39, 2.39	-	-	-	-	34.68	31.68	-	-	-	-
**N26**	125.81	112.63	-	9.39	4.28	2.96, 2.68	-	6.85, 7.53	-	-	54.71	37.29	-	-	-	-	-
**R27**	107.69	-	125.03	8.40	3.79	2.11, 2.11	1.54, 1.54	3.17, 3.17	7.10	-	58.48	27.39	27.94	43.46	-	-	-
**K28**	119.36	-	-	7.66	4.77	1.66, 1.66	1.35, 1.37	1.62, 1.62	2.98, 2.98	-	-	36.01	24.84	29.12	42.31	-	-
**U29**	122.20	-	-	8.76	4.76	2.68, 2.68	-	-	-	-	56.98	29.23	-	-	-	-	278.0
**K30**	127.77	-	-	9.20	4.73	1.84, 1.84	1.35, 1.35	1.70, 1.70	-	-	54.67	33.89	24.81	29.09	-	-	-
**U31**	127.34	-	-	9.01	5.33	3.20, 2.92	-	-	-	-	55.00	30.35	-	-	-	-	250.9
**T32**	114.41	-	-	8.80	4.66	4.24	1.04	-	-	-	-	71.71	21.70	-	-	-	-
**N33**	117.02	112.20	-	8.72	4.29	3.07, 2.92	-	7.43, 6.86	-	-	-	37.34	-	-	-	-	-
**U34**	-	-	-	8.45	4.78	3.53, 3.12	-	-	-	-	57.58	34.49	-	-	-	-	346.3
**K35**	128.78	-	-	8.19	4.19	1.75, 1.87	1.44, 1.63	1.63, 1.75	2.93, 2.93	-	57.65	33.85	25.04	29.11	42.17	-	-

Bruker pulse sequence code of the refocused and X-decoupled CPMG-HSQMBC experiment

```
;ek_ti_cpmg_hsqmbc_dc
;
;refocused and X-decoupled CPMG-HSQMBC pulse sequence
;2D 1H-X correlation via double inept transfer
;phase sensitive using Echo/Antiecho gradient selection
;using CPMG for polarization transfer to avoid evolution of J(HH)
;using composite 1H and X 180 pulses in CPMG
;with gradient z,z filter purge pulse between last 90 degree pulse pair
;with X-decoupling during acquisition
;
;This pulse sequence is part of the manuscript submitted.
;
;Further relevant publications:
;K.E. Kövér, G. Batta, K. Fehér, J. Magn. Reson. 2006, 181, 89-97.
;S. Boros, K.E. Kövér, Magn. Reson. Chem. 2011, 49, 106-110.
;I. Timári, T.Z. Illyés, R.W. Adams, M. Nilsson, L. Szilágyi, G.A. Morris,
;K.E. Kövér, Chem. Eur. J. 2015, 21, 3472-3479.
;
;Bruker Avance II version

#include <Avance.incl>
#include <Grad.incl>


"p2=p1*2"
"p4=p3*2"
"d0=3u"
"d11=30m"
"d13=3u"
"d7=d13+p16+d16+4u"


"d20=p16+d16+p2+d0*2"
"l3=(td1/2)"
"in0=inf1/2"


1 ze
  d11 pl12:f2
2 d1 do:f2
  d11
3 d11 pl1:f1
4 (p1 ph1)

5  d15 pl2:f2                  ;CPMG-sequence for polarization transfer
;with XY-16 phase cycle
  (p1 ph20) (p3 ph20):f2
   3u
  (p2 ph21) (p4 ph21^):f2      ;^ increment phase pointer of ph21
   3u
  (p1 ph20) (p3 ph20^):f2      ;^ increment phase pointer of ph20 -
;composite 1H and X pulses
   d15                         ;d15=140-150us
   lo to 5 times l1            ;p1 should be calibrated to p1=p3 at pl1!!!
                               ;l1=multiple of 16
                        ;long-range coupling evolution = (2*d15+2*p4+6)*l1
```

```
  (p1 ph2)
  d13 UNBLKGRAD
  p16:gp3                   ;gpz3=19 purging
  d16
  (p3 ph3):f2
  d0
  (p2 ph5)
  d0
  p16:gp1                   ;gpz1=80 for echo-antiecho coherence selection
  d16

  (p3 ph14):f2              ;comp. X 180 pulse
  (p4 ph4):f2
  (p3 ph14):f2

  d20
  (p3 ph4):f2
  d13
  p16:gp4                   ;gpz4=10 purging
  d16
  (p1 ph1)

6  d15                        ;CPMG-sequence for polarization transfer
;with XY-16 phase cycle
  (p1 ph20) (p3 ph20):f2
   3u
  (p2 ph21) (p4 ph21^):f2     ;^ increment phase pointer of ph21
   3u
  (p1 ph20) (p3 ph20^):f2     ;^ increment phase pointer of ph20 -
;composite 1H and X pulses
   d15                        ;d15=140-150us
   lo to 6 times l1           ;p1 should be calibrated to p1=p3 at pl1!!!
                              ;l1=multiple of 16
                         ;long-range coupling evolution = (2*d15+2*p4+6)*l1

  d7
  (p2 ph1)
  d13
  p16:gp2*EA                  ;gpz2=20.1 for 13C and 15.26 for 77Se for
;echo-antiecho coherence selection
  d16 pl12:f2
  4u BLKGRAD

  go=2 ph31 cpd2:f2
  d1 do:f2 wr #0 if #0 zd
  d11 igrad EA
  lo to 3 times 2
  d11 id0
  lo to 4 times l3
exit


ph1=0
ph2=1
ph20=1 2 1 2 2 1 2 1 3 0 3 0 0 3 0 3
ph21=0 1 0 1 1 0 1 0 2 3 2 3 3 2 3 2
ph3=0 2
ph4=0 0 0 0 2 2 2 2
ph14=1 1 1 1 3 3 3 3
ph5=0 0 2 2
ph31=0 2 0 2 2 0 2 0
```

```
;pl1 : f1 channel - power level for pulse (default)
;pl2 : f2 channel - power level for pulse (default)
;pl12: f2 channel - power level for CPD/BB decoupling
;p1 : f1 channel -  90 degree high power pulse
;p2 : f1 channel - 180 degree high power pulse
;p3 : f2 channel -  90 degree high power pulse
;p4 : f2 channel - 180 degree high power pulse
;p16: homospoil/gradient pulse
;d0 : incremented delay                  [3 usec]
;d1 : relaxation delay; 1-5 * T1
;d7: =d13+p16+d16+4u
;d11: delay for disk I/O                 [30 msec]
;d13: short delay                        [3 usec]
;d15: interpulse delay                   [140-150 usec]
;d16: delay for homospoil/gradient recovery
;d20: =p16+d16+p2+d0*2
;l1: loop for CPMG
;NS: number of scans
;DS: number of dummy scans, >= 128!
;FnMODE1: Echo-Antiecho
;cpd2: decoupling according to sequence defined by cpdprg2
;pcpd2: f2 channel - 90 degree pulse for decoupling sequence
;gpz1: 80%
;gpz2: 20.1% for C-13, 15.26% for Se-77
;gpz3: purging gradient (~19%)
;gpz4: purging gradient (~10%)
;
;use gradient files:
;gpnam1: SINE.100
;gpnam2: SINE.100
;gpnam3: SINE.100
;gpnam4: SINE.100
```
